# Transcriptome analysis of bronchoalveolar lavage fluid from children with severe *Mycoplasma pneumoniae* pneumonia reveals novel gene expression and immunodeficiency

**DOI:** 10.1186/s40246-017-0101-y

**Published:** 2017-03-16

**Authors:** Kuo Wang, Man Gao, Mingyue Yang, Fanzheng Meng, Deli Li, Ruihua Lu, Yan Wang, Huadong Zhuang, Mengyao Li, Genhong Cheng, Xiaosong Wang

**Affiliations:** 1 Institute of Translational Medicine, the First Hospital of Jilin University, Changchun, 130061 China; 2 Department of Pediatrics, the First Hospital of Jilin University, Changchun, 130021 China; 3 Bethune Medical School of Jilin University, Changchun, 130021 China; 4 Department of Microbiology, Immunology and Molecular Genetics, University of California Los Angeles, Los Angeles, CA 90095 USA

**Keywords:** Severe *Mycoplasma pneumoniae* pneumonia, Children, Bronchoalveolar lavage fluid, Transcriptome sequencing, Gene expression profile, Alternative splicing

## Abstract

**Background:**

A growing number of severe *Mycoplasma pneumoniae* pneumonia (MPP) cases have been reported recently. However, the pathogenesis of severe MPP is not clear. In the current study, transcriptome sequencing was used to identify gene expression and alternative splicing profiles to provide insights into the pathogenesis of severe MPP.

**Methods:**

RNAs of bronchoalveolar lavage fluid (BALF) samples from three severe MPP children and three mild MPP children were analyzed respectively by deep sequencing followed by computational annotation and quantification.

**Results:**

The gene expression analysis revealed 14 up-regulated and 34 down-regulated genes in severe MPP children comparing to mild MPP children. The top 10 most up-regulated genes were *IGHV1-69*, *CH17-472G23.1*, *ATP1B2*, *FCER2*, *MUC21*, *IL13*, *FCRLB*, *CLEC5A*, *FAM124A*, and *INHBA*. The top 10 most down-regulated genes were *OSTN-AS1*, *IL22RA2*, *COL3A1*, *C1orf141*, *IGKV2-29*, *RP11-731F5.2*, *IGHV4-4*, *KIRREL*, *DNASE1L3*, and *COL6A2*. Clustering analysis revealed similar expression pattern of *CLEC5A*, *IL13*, *FCER2*, and *FLT1*. Kyoto Encyclopedia of Genes and Genomes (KEGG) pathway enrichment analyses revealed changes related to primary immunodeficiency in severe MPP children comparing to mild MPP children; the pathway involves *CD19*, *TNFRSF13C*, *CD79A*, and *AICDA* genes. Among the differentially expressed genes, significant alternative splicing events were found in *FCER2* and *FCRLA*.

**Conclusions:**

The current study on RNA sequencing provides novel insights into the pathogenesis of severe MPP in terms of gene expression and alternative splicing. The up-regulation of *IL13*, *FCER2*, *FLT1*, and *CLEC5A* and the down-regulation of *CD79A*, *AICDA*, *CD19*, and *TNFRSF13C* may contribute to the pathogenesis of severe MPP. The differential expressions of *FCER2* and *FCRLA* could be due to their alternative splicing.

**Electronic supplementary material:**

The online version of this article (doi:10.1186/s40246-017-0101-y) contains supplementary material, which is available to authorized users.

## Background

*Mycoplasma pneumoniae* pneumonia (MPP), as a common community-acquired pneumonia, counts for 20 to 40% of children pneumonia and may reach 50 to 80% during the time of local outbreak [1, 2]. MPP is usually described as mild and self-limited; however, more and more severe or even fatal cases of MPP with severe complications such as pulmonary necrosis and chronic interstitial fibrosis have been reported recently [3–5]. Macrolide-resistant and excessive immunological inflammation are also commonly found in severe MPP [6]. Therefore, it is essential for pediatricians to recognize severe MPP early, treat it promptly, and prevent the progression of the disease effectively. However, the mechanism and etiology of severe MPP are largely unknown.

Based on published hypotheses, severe MPP is considered as a hyper-immune response that originates from repeated or longer lasting childhood MP infections in the lung [7]; further, severe MPP can be an overactive innate immune response such as macrophage activation via heterodimerization of Toll-like receptors two and six of the bronchoepithelial cells to *M. pneumoniae* lipoproteins [8]. With ELISA and real-time quantitative PCR techniques, researchers have found that the cell-mediated immune response plays an important role in the pathogenesis of MPP [9–11] but the role of humoral-mediated immune response in mild and severe MPP is still unclear.

High-throughput RNA sequencing technology, so called next-generation sequencing, revolutionarily enhanced our understanding on the complexity of eukaryotic transcriptome [12, 13]. It has several key advantages including being independent on the predetermined genome sequences, highly accurate in detecting gene expression with very wide dynamic detection ranges with low background. Thus, RNA sequencing is not only useful to precisely determine gene expression profiles but also particularly powerful to detect novel transcription variants via alternative splicing [12].

In the present study, we observed the transcriptome of bronchoalveolar lavage fluid (BALF) from children with mild MPP and severe MPP. The large sum of novel information on the gene expression profiles as well as novel transcripts through alternative splicing would provide not only insights into the pathogenesis of severe MPP but also as basis for the development of biomarkers and therapeutic targets.

## Methods

### Study subjects

The current study was conducted at the First Hospital of Jilin University (Changchun City, Jilin Province, People’s Republic of China). Six newly diagnosed children (three male and three female) with acute stage of MPP admitted to our hospital were recruited [see Additional file 1: Table S1]. All of the children enrolled in this study had no recurrent severe or unusual infections and had no inflammatory disorders or autoimmunity. Therefore, based on the published diagnostic criteria, they had no history of common variable immunodefiency (CVID) [14]. After admission to our hospital, the levels of immunoglobulins in the blood of these children had been examined; the levels of IgG, IgA, and IgM had been found within normal range published for children [see Additional file 2: Figure S1] [15]. Lymphocyte profiles in the peripheral blood of these children had also been examined, the cell numbers and percentage of T cells, B cells, and natural killer cells had been found within normal range [see Additional file 3: Table S2] [15]. Therefore, the enrolled children had been excluded from having CVID, autosomal recessive agammaglobulinemia [15], or high IgM syndrome [16]. All children did not have untreated metabolic/congenital systemic diseases. The diagnosis of pneumonia was based on clinical manifestations (cough, fever, dry or productive sputum, dyspnea, abnormal breath sound, radiological pulmonary abnormalities). The diagnosis of *Mycoplasma pneumoniae* (MP) infection was based on positive results of serologic test (MP-IgM test ≥1:40) and positive results of MP DNA (>500 copy/L) in BALF with real-time quantitative PCR. MP was the only pathogen identified in all the MPP subjects. The mild and severe community-acquired pneumonia was defined based on the criteria described [17, 18]. Mild group was defined as fever <38.5 °C at any age, tachypnea but respiratory rate <70 breaths/min at age <3 years old or <50 breaths/min at age ≥3 years old, normal food-intake, and no dehydration. Severe group was defined as fever ≥38.5 °C at any age, breathless with respiratory rate ≥70 breaths/min at age <3 years old or ≥50 breaths/min at age ≥3 years old (excluding the reasons of fever and cry), cyanosis, marked retractions, anorexia, and dehydration.

The written informed consents were obtained by care givers of all children. The study was approved by the Institutional Medical Ethics Review Board of the First Hospital of Jilin University in compliance with the Declaration of Helsinki.

### Bronchoscopy and bronchoalveolar lavage

Following the guidelines described previously [17], flexible fiber optic bronchoscopy with bronchoalveolar lavage (BAL) was performed within 3 days after the admission. Both groups received similar supportive and symptomatic treatment, including sputum aspiration, nebulization, and fluid therapy. Corticosteroid and the other immune regulation agents were not permitted before bronchoscopy. A 2.8-mm pediatric flexible bronchoscope (Olympus BF-XP60, New Hyde Park, NY) or a 4.0-mm flexible bronchoscope (Olympus P-260, New Hyde Park, NY) was used for children depending on their age and body weight. All of the enrolled subjects had indications for bronchoscopy and BAL: radiologically proven large pulmonary lesions (including atelectasis and consolidation of lung fields). Supplemental oxygen was administered during the procedure. Transcutaneous oxygen saturation and pulse rate (Masimo Radical-7 pulse oximetry, Masimo, CA) were continuously monitored during the bronchoscopy.

Intravenous injection of midazolam (0.1–0.15 mg/kg) was used for sedation; aerosolized lidocaine spraying on the throat insertion was performed 5–10 min before bronchoscope for throat local anesthesia; dripping 2% lidocaine through flexible bronchoscopy was used for the topical anesthesia of the upper and lower airways. BAL was performed in an area most prominently affected based on the chest radiology (MPP groups) by gently wedging the tip of the Bronchoscope in a segmental or subsegmental bronchus. 1 ml/kg sterile saline was instilled through the instrumentation channel. The BALF was gently aspirated and collected in a sterile container and immediately centrifuged. The pallet was resuspended in TRIzol (Life Technologies, CA, USA) and stored in −80 °C freezer. The composition of the nucleated cells in the BALF was counted; results were shown in Additional file 4: Table S3.

### RNA preparation and sequencing

Total RNA was extracted using TRIzol according to the manufacturer’s instructions. RNA degradation and contamination were monitored on 1% agarose gels. RNA purity was checked using the NanoPhotometer spectrophotometer (IMPLEN, CA, USA). RNA concentration was measured using Qubit RNA Assay Kit in Qubit 2.0 Flurometer (Life Technologies, CA, USA). RNA integrity was assessed using the RNA Nano 6000 Assay Kit of the Bioanalyzer 2100 system (Agilent Technologies, CA, USA).

A total amount of 3 μg RNA per sample was used as input material for the RNA sample preparation. Sequencing libraries were generated using NEBNext® Ultra™ RNA Library Prep Kit for Illumina® (NEB, USA) following the manufacturer’s recommendations, and index codes were added to attribute sequences to each sample. The clustering of the index-coded samples was performed on a cBot Cluster Generation System using TruSeq PE Cluster Kit v3-cBot-HS (Illumia) according to the manufacturer’s instructions. After cluster generation, the library preparations were sequenced on an Illumina Hiseq platform and 125 bp/150 bp paired-end reads were generated.

### Sequencing data analysis

Raw data (raw reads) of fastq format were firstly processed through in-house perl scripts, and clean data (clean reads) were obtained. Index of the reference genome was built using Bowtie v2.2.3, and paired-end clean reads were aligned to the reference genome using TopHat v2.0.12. Only uniquely and properly mapped read pairs were used for further analysis. The differentially expressed genes between BALF samples were identified using the DESeq R package (1.18.0) [19]. Differentially expressed genes were defined as those with changes of at least twofold between samples. The resulting *p* values were adjusted using the Benjamini and Hochberg approach for controlling the false discovery rate (FDR). Genes with an adjusted *p* value <0.05 found by DESeq were assigned as differentially expressed. Protein functional classification of differentially expressed genes was performed using the PANTHER classification system [20]. KOBAS software was used to test the statistical enrichment of differential expression genes in KEGG pathways. The significance of enriched KEGG pathways were determined by corrected *p* value <0.05. Cufflinks v2.1.1 Reference Annotation Based Transcript (RABT) assembly method was used to construct and identify both known and novel transcripts from TopHat alignment results. The analysis of alternative splicing events was performed using MATS and IGV software [21]; alternative splicing events were classified to five basic types by software Asprofile v1.0. The differences in alternative splicing of genes were considered significant with a cutoff of 5% FDR.

## Results

### RNA sequencing results

Total RNA was extracted from six BALF samples of children with severe MPP or mild MPP [see Additional file 1: Table S1]. Then, mRNAs from each sample were sequenced. After the removal of adaptor sequences, ambiguous reads and low-quality reads, about 40–70 million pairs of clean read, were generated for each sample (Table 1). The percentage of reads mapped to the forward chain was equal to that mapped to the reverse chain. When compared with the reference sequence of the Genome Reference Consortium GRCh37/hg19, more than 85% of total read pairs were uniquely mapped on the human genome (Table 1). A correlation matrix shown a high consistency of measurements within each group, *R* ^2^ > 0.8 [see Additional file 5: Figure S2A]. Principal component analysis (PCA) was carried out to assess the clustering nature of these samples. Samples of each group had been clustered together; data shown good repeatability and correlation [see Additional file 5: Figure S2B].

**Table 1:** Summary of RNA sequencing read mapping result

Sample number	Mild 202	Mild 224	Mild 247	Severe 177	Severe 223	Severe 324
Total reads	55492664	47186542	64792680	68897254	42136856	46318894
Total mapped	48535137 (87.46%)	40517410 (85.87%)	56013052 (86.45%)	60481397 (87.78%)	36565361 (86.78%)	40224088 (86.84%)
Multiple mapped	844736 (1.52%)	614681 (1.30%)	797662 (1.23%)	881129 (1.28%)	543196 (1.29%)	630869 (1.36%)
Uniquely mapped	47690401 (85.94%)	39902729 (84.56%)	55215390 (85.22%)	59600268 (86.51%)	36022165 (85.49%)	39593219 (85.48%)
Reads map to “+”	23844413 (42.97%)	19967766 (42.32%)	27594117 (42.59%)	29790936 (43.24%)	18008541 (42.74%)	19797319 (42.74%)
Reads map to “−”	23845988 (42.97%)	19934963 (42.25%)	27621273 (42.63%)	29809332 (43.27%)	18013624 (42.75%)	19795900 (42.74%)
Non-splice reads	30580304 (55.11%)	22475106 (47.63%)	34530918 (53.29%)	37577003 (54.54%)	20733762 (49.21%)	24205574 (52.26%)
Splice reads	17110097 (30.83%)	17427623 (36.93%)	20684472 (31.92%)	22023265 (31.97%)	15288403 (36.28%)	15387645 (33.22%)

Total reads: The number of sequence after sequencing data filtering

Total mapped: The number of sequence which can map to the genome

Multiple mapped: The number of sequence which have multiple location on the reference sequencing

Uniquely mapped: The number of sequence which have single location on the reference sequencing

Reads map to “+”: The statistics of sequence that were mapped on the “+” chain of the genome

Reads map to “−”: The statistics of sequence that were mapped on the “−” chain of the genome

### Identification and classification of differentially expressed genes between severe MPP and mild MPP

**Fig. 1:**
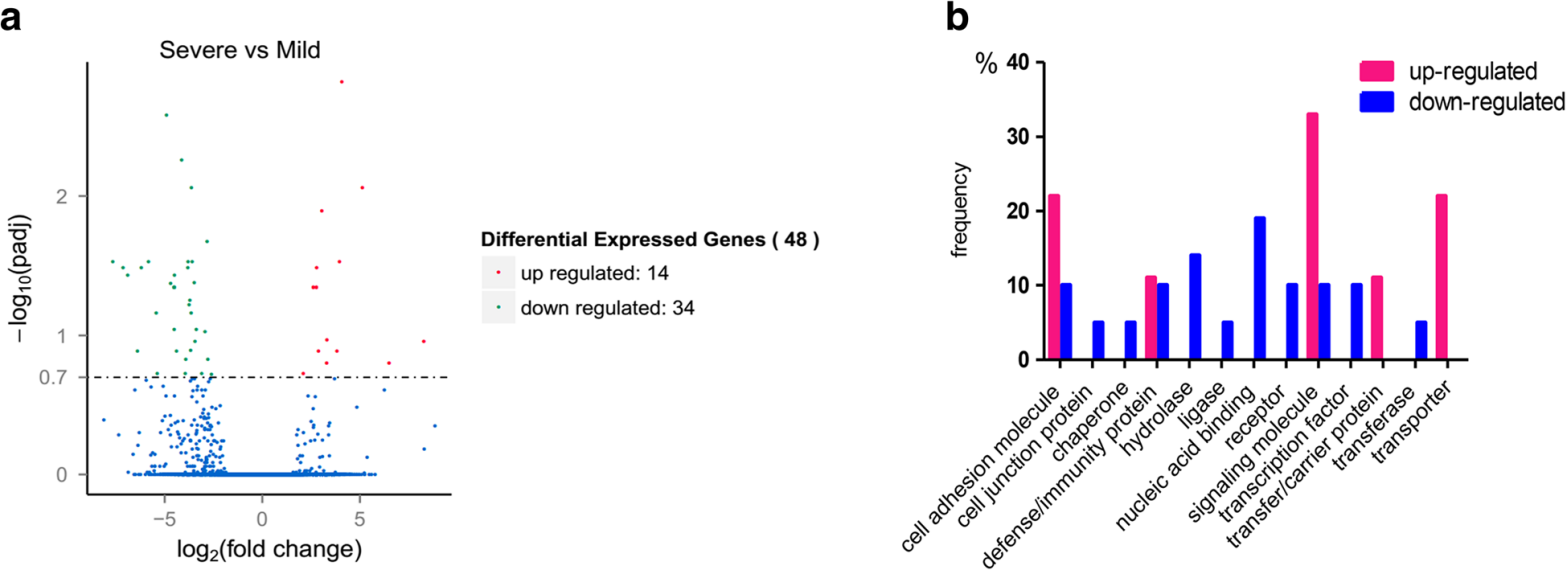
Differentially expressed genes between the severe MPP group and mild MPP group in BALF. **a** Volcano plot of genes differentially expressed between the severe MPP group and mild MPP group. Each point represents one gene that is detectable in both groups. The *red points* represent significantly up-regulated genes; the *green points* represent significantly down-regulated genes. **b** Protein functional classification of differentially expressed genes has been performed using the PANTHER tool between the severe MPP group and mild MPP group. The protein category is shown on *x*-axis and the gene frequency is shown on *y*-axis

Totally, 48 differentially expressed genes were identified between the severe MPP group and mild MPP group (Fig. 1a). The 14 up-regulated genes listed in Table 2 were *IGHV1-69*, *CH17-472G23.1*, *ATP1B2*, *FCER2*, *MUC21*, *IL13*, *FCRLB*, *CLEC5A*, *FAM124A*, *INHBA*, *FLT1*, *APOL4*, and two novel transcripts. These 14 up-regulated genes included cytokine (*IL13*), immunoglobin Fc receptors (*FCER2* and *FCRLB*), and inflammatory response regulator (*CLEC5A*). The top 20 most down-regulated genes were *OSTN-AS1*, *IL22RA2*, *COL3A1*, *C1orf141*, *IGKV2-29*, *RP11-731F5.2*, *IGHV4-4*, *KIRREL*, *DNASE1L3*, *COL6A2*, *COL6A1*, *FCRL4*, *HTRA3*, *TCL1A*, *RP11-356K23.1*, *PLD4*, *DKK3*, *UBE2QL1*, *KLRB1*, and *MS4A1* (Table 3).

**Table 2:** Up-regulated genes of the severe MPP group comparing to the mild MPP group

Gene ID	Associated gene name	log_2_foldchange	padj	Description
ENSG00000104921	*FCER2*	4.081288	0.001514	Fc fragment of IgE, low affinity II, receptor for (CD23)
ENSG00000129244	*ATP1B2*	5.123508	0.008687	ATPase, Na+/K+ transporting, beta 2 polypeptide
ENSG00000258227	*CLEC5A*	3.046087	0.012798	C-type lectin domain family 5 member A
Novel00326	–	3.957912	0.029750	–
ENSG00000122641	*INHBA*	2.792227	0.032804	Inhibin beta A
ENSG00000102755	*FLT1*	2.773680	0.044977	Fms-related tyrosine kinase 1
Novel00056	–	2.612021	0.044977	–
ENSG00000169194	*IL13*	3.320997	0.107345	Interleukin 13
ENSG00000211973	*IGHV1-69*	8.269132	0.110361	Immunoglobulin heavy variable 1–69
ENSG00000150510	*FAM124A*	2.878502	0.130273	Family with sequence similarity 124 member A
ENSG00000204544	*MUC21*	3.830138	0.130273	Mucin 21, cell surface associated
ENSG00000162746	*FCRLB*	3.307526	0.157765	Fc receptor-like B
ENSG00000274642	*CH17-472G23.1*	6.496741	0.157765	–
ENSG00000100336	*APOL4*	2.103424	0.186854	Apolipoprotein L4

*log*_*2*_ *foldchange* log_2_ (severe MPP/mild MPP), *padj* adjusted *p* value, *Novel* novel gene

**Table 3:** Down-regulated genes of the severe MPP group comparing to the mild MPP group

Gene ID	Associated gene name	log_2_foldchange	padj	Description
ENSG00000163687	*DNASE1L3*	−4.90655	0.002613	Deoxyribonuclease I-like 3
ENSG00000166428	*PLD4*	−4.12788	0.005539	Phospholipase D family member 4
ENSG00000269404	*SPIB*	−3.62528	0.008687	Spi-B transcription factor (Spi-1/PU.1 related)
ENSG00000081059	*TCF7*	−2.83313	0.021184	Transcription factor 7 (T cell specific, HMG-box)
ENSG00000156738	*MS4A1*	−3.80125	0.029750	Membrane-spanning 4-domains, subfamily A, member 1
ENSG00000159958	*TNFRSF13C*	−3.59110	0.029750	Tumor necrosis factor receptor superfamily member 13C
ENSG00000233308	*OSTN-AS1*	−7.65241	0.029750	OSTN antisense RNA 1
ENSG00000253364	*RP11-731F5.2*	−5.82530	0.029750	–
ENSG00000111796	*KLRB1*	−3.80851	0.032804	Killer cell lectin-like receptor subfamily B, member 1
ENSG00000164485	*IL22RA2*	−7.13138	0.032804	Interleukin 22 receptor subunit alpha 2
ENSG00000253998	*IGKV2-29*	−6.19209	0.032804	Immunoglobulin kappa variable 2–29 (gene/pseudogene)
ENSG00000168542	*COL3A1*	−6.89272	0.036862	Collagen, type III, alpha 1
ENSG00000170801	*HTRA3*	−4.50354	0.036862	HtrA serine peptidase 3
Novel00339	–	−3.48127	0.041780	–
ENSG00000142173	*COL6A2*	−4.69524	0.042554	Collagen, type VI, alpha 2
ENSG00000100721	*TCL1A*	−4.49274	0.044977	T cell leukemia/lymphoma 1A
ENSG00000142156	*COL6A1*	−4.52065	0.044977	Collagen, type VI, alpha 1
ENSG00000111732	*AICDA*	−3.71025	0.055876	Activation-induced cytidine deaminase
ENSG00000164692	*COL1A2*	−3.75418	0.059895	Collagen, type I, alpha 2
ENSG00000105369	*CD79A*	−3.63713	0.069048	CD79a molecule
ENSG00000276775	*IGHV4-4*	−5.43537	0.069048	Immunoglobulin heavy variable 4-4
ENSG00000080573	*COL5A3*	−3.37399	0.090838	Collagen, type V, alpha 3
ENSG00000163518	*FCRL4*	−4.51056	0.090838	Fc receptor-like 4
ENSG00000196092	*PAX5*	−2.93710	0.093937	Paired box 5
ENSG00000177455	*CD19*	−3.44060	0.110361	CD19 molecule
ENSG00000136573	*BLK*	−3.67317	0.128424	BLK proto-oncogene, Src family tyrosine kinase
ENSG00000203963	*C1orf141*	−6.39692	0.130273	Chromosome 1 open reading frame 141
ENSG00000258752	*RP11-356K23.1*	−4.39265	0.130273	–
ENSG00000167483	*FAM129C*	−2.78386	0.148367	Family with sequence similarity 129 member C
ENSG00000215218	*UBE2QL1*	−3.91473	0.149275	Ubiquitin conjugating enzyme E2Q family-like 1
ENSG00000050165	*DKK3*	−3.95056	0.186854	Dickkopf WNT signaling pathway inhibitor 3
ENSG00000132185	*FCRLA*	−3.11003	0.186854	Fc receptor-like A
ENSG00000183853	*KIRREL*	−5.37583	0.186854	Kin of IRRE like (drosophila)
ENSG00000117215	*PLA2G2D*	−2.60098	0.191201	Phospholipase A2 group IID

*log*_*2*_ *foldchange* log_2_ (severe MPP/mild MPP), *padj* adjusted *p* value, *Novel* novel gene

Protein functional classification of differentially expressed genes between severe MPP and mild MPP was performed. As a result, the 48 differentially expressed genes were divided into 13 different classes of protein (Fig. 1b). The expression levels of genes classified as signaling molecule (*CLEC5A*, *IL13*, *INHBA*), transporter (*ATP1B2*), and transfer/carrier protein (*APOL4*) significantly increased in the severe MPP group comparing to the mild MPP group [see Additional file 6: Table S4]. On the other hand, genes in the categories of nucleic acid binding molecule (*AICDA*, *PAX5*, *SPIB*, *TCF7*), genes encodes defense/immunity protein (*IL22RA2*, *CD79A*), hydrase (*PLA2G2D*, *HTRA3*, *AICDA*), receptor (*MS4A1*, *IL22RA2*), and transcription factor (*PAX5*), were predominantly expressed in the mild MPP group. Among the cell adhesion molecules, *FCER2* and *FCRLB* were up-regulated in the severe MPP group; *FCRLA* and *FCRL4* were up-regulated in the mild MPP group.

**Fig. 2:**
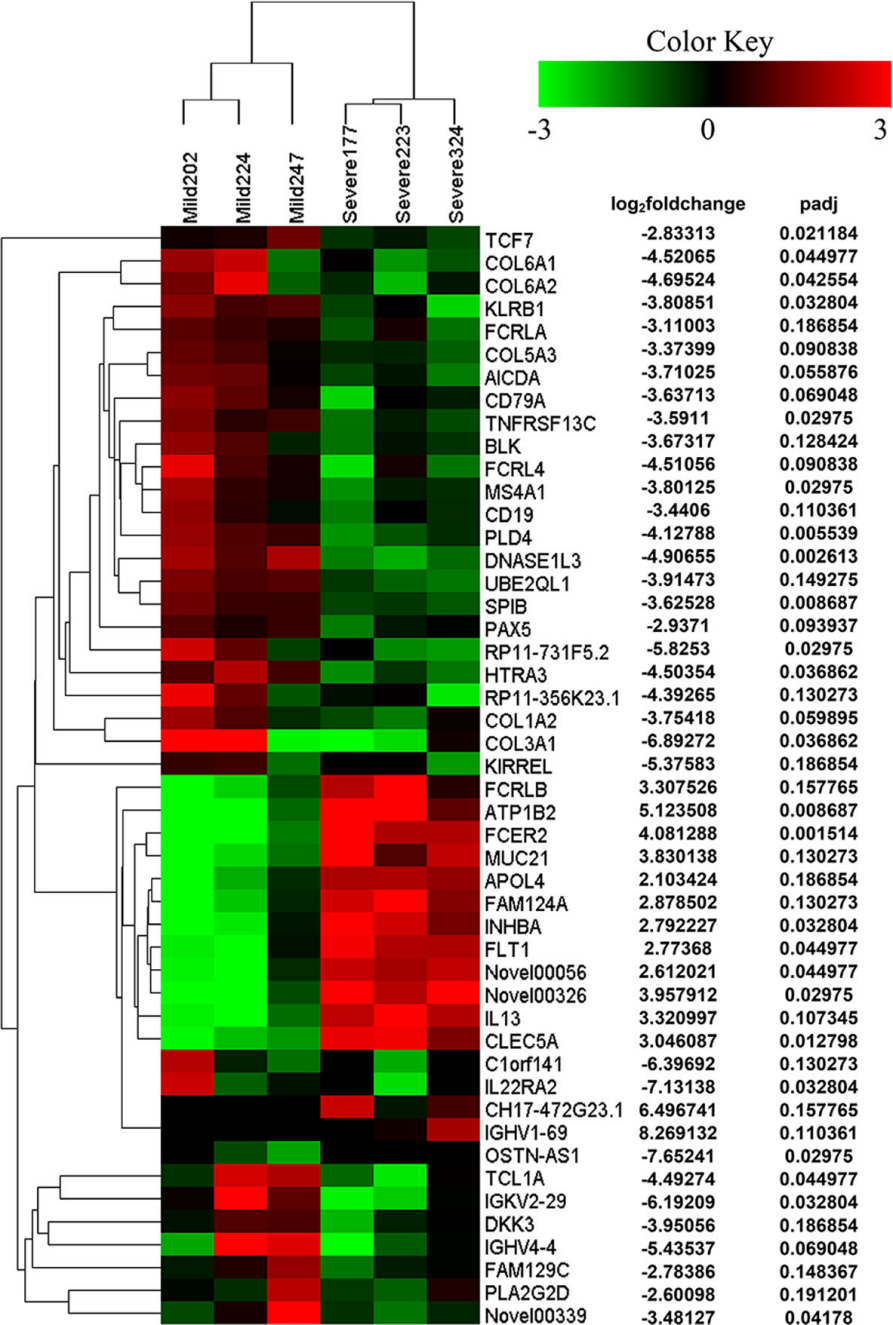
Cluster of 48 genes showing representative expression patterns between the severe MPP group and mild MPP group. All of the genes that are differentially expressed between the severe MPP group and mild MPP group by log_2_ fold change >2 or <−2, adjusted *p* value <0.2, have been selected

The clustering analysis of differentially expressed genes indicated that *FCER2*, *FLT1*, *IL13*, and *CLEC5A* were up-regulated in the severe MPP group comparing to the mild MPP group (Fig. 2). Similar up-regulation or down-regulation patterns among these genes were identified, which may indicate related local protein functions of these genes under MP infection in children.

**Fig. 3:**
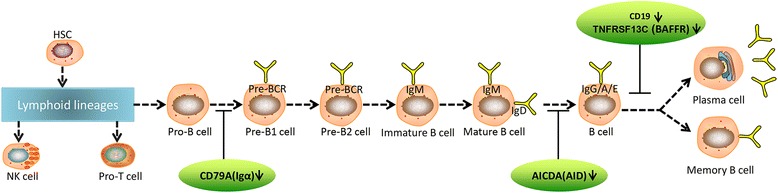
Model diagram showing primary immunodeficiency. Hematopoietic stem cell (HSC)-derived lymphoid progenitor cells develop into progenitor (pro)-B cells, Pre-B1 cell, Pre-B2 cell, immature B cell, mature B cell, B cell, memory B cell, and plasma cell. The decreased expression of *CD79A* inhibits the differentiation of Pro-B cell into Pre-B1 cell; the decreased expression of *AICDA* inhibits the differentiation of mature B cell into B cell; the decreased expression of *CD19* and *TNFRSF13C* inhibits the differentiation of mature B cell into memory B cell and plasma B cell

Functional annotation of KEGG pathway enrichment analysis revealed changes related to primary immunodeficiency in the BALF of severe MPP children, which was associated with the down-regulation of *CD19*, *TNFRSF13C*, *CD79A*, and *AICDA* genes (Table 4). *CD19*, *TNFRSF13C*, *CD79A*, and *AICDA* were involved in the B cell differentiation process; down-regulation of these genes may restrain B cell maturation and antibody production (Fig. 3) [22]. Furthermore, *COL6A1*, *COL6A2*, *COL5A3*, *COL3A1*, and *COL1A2* genes were involved in the pathways including protein digestion and absorption, ECM-receptor interaction, P13K-Akt signaling, and focal adhesion; these molecules were found down-regulated in the severe MPP group comparing to the mild MPP group (Table 4). *COL6A1*, *COL6A2*, *COL5A3*, *COL3A1*, and *COL1A2* belong to the collagen super family, which play a role in maintaining the integrity of various tissues including the lung [23]. *FLT1*, which encoded a member of vascular endothelial growth factor receptor (VEGFR) family, was also found significantly increased in the severe MPP group comparing to the mild MPP group in the P13K-Akt signaling and focal adhesion pathways (Tables 2 and 4).

**Table 4:** KEGG pathway enrichment

Term	ID	Adjusted *p* value	Input	KEGG_ID/KO
Protein digestion and absorption	hsa04974	0.000305	*COL6A1| COL6A2| ATP1B2| COL5A3| COL3A1| COL1A2*	hsa:1291|hsa:1292|hsa:482|hsa:50509|hsa:1281|hsa:1278|
Primary immunodeficiency	hsa05340	0.001040	*CD19| TNFRSF13C| CD79A| AICDA*	hsa:930|hsa:115650|hsa:973|hsa:57379|
ECM-receptor interaction	hsa04512	0.001350	*COL6A2| COL5A3| COL6A1| COL1A2| COL3A1*	hsa:1292|hsa:50509|hsa:1291|hsa:1278|hsa:1281|
PI3K-Akt signaling pathway	hsa04151	0.003493	*COL6A1| TCL1A| COL6A2| CD19| COL5A3| COL3A1| COL1A2| FLT1*	hsa:1291|hsa:8115|hsa:1292|hsa:930|hsa:50509|hsa:1281|hsa:1278|hsa:2321|
Focal adhesion	hsa04510	0.005512	*COL6A1| COL6A2| COL5A3| COL3A1| COL1A2| FLT1*	hsa:1291|hsa:1292|hsa:50509|hsa:1281|hsa:1278|hsa:2321|

### Alternative splicing events between severe MPP and mild MPP

More than 90% of human genes are alternatively spliced through different types of splicing [24]. MATS analysis of the RNA sequencing data significantly revealed 1500 differential alternative splicing events with a cutoff of 5% FDR (Table 5), more than half (50.5%) of them belong to the skipped exon type. Among the differentially expressed genes between the severe MPP group and mild MPP group, *FECR2* and *FCRLA* were identified to have significantly alternative splicing [see Additional file 7: Table S5, Additional file 8: Table S6, Additional file 9: Table S7, Additional file 10: Table S8, and Additional file 11: Table S9].

**Table 5:** Summary of the differential alternative splicing event analysis

	Skipped exon	Retained intron	Mutual exclusive exon	Alternative 5′ splicing sites	Alternative 3′ splicing sites
Number of total alternative splicing events (genes)	44856 (11195)	3772 (2353)	6862 (3327)	2793 (2103)	4374 (2874)
Percentage of total alternative splicing event (%)	71.6	6.0	10.9	4.5	7.0
Number of differential alternative splicing events (up-regulation/down-regulation)	758 (398:360)	181 (107:74)	315 (193:122)	101 (74:27)	145 (86:59)
Percentage of total differential alternative splicing event (%)	50.5	12.1	21	6.7	9.7

**Fig. 4:**
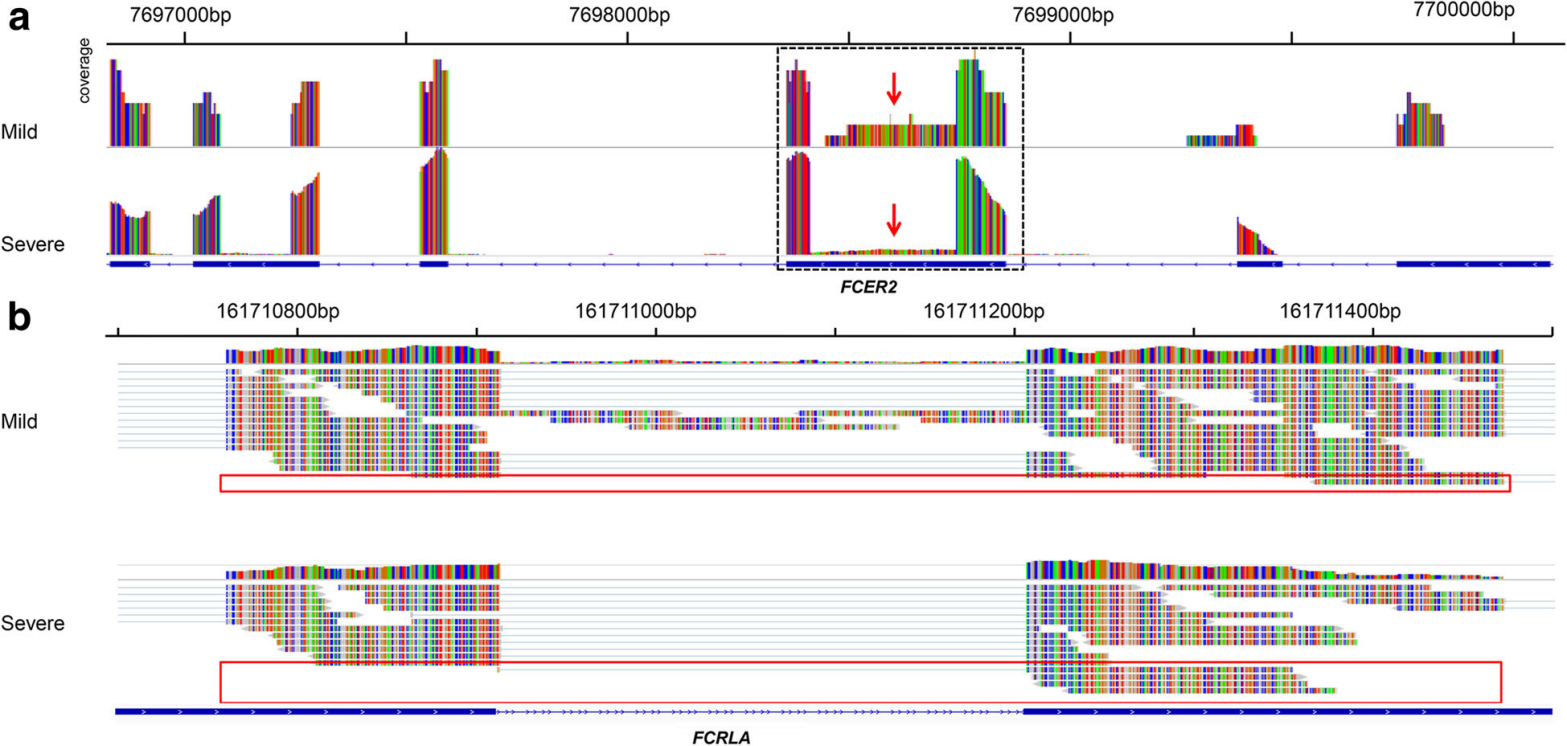
Differential alternative splicing of *FCER2* and *FCRLA*. **a** Read distribution plot for *FCER2* with differential isoform expression due to retained intron in the mild MPP group is shown. The *black box* and *red arrows* indicated the location of the retained intron. **b** Read distribution plot for *FCRLA* with differential isoform expression due to mutually exclusive exon is shown. The ratio of mutually exclusive exon happened in the severe MPP group is compared with that in the mild MPP group

*FCER2* locates on chromosome 19p13.2 (chr 19:7,688,758-7,702,146); its encoding protein is a B cell specific antigen, which is a low-affinity receptor for IgE. This protein plays essential roles in B cell growth, differentiation, and regulation of IgE production. It also exists as a soluble secreted form and acts as a potent mitogenic growth factor. Retained intron (RI) was identified in *FCER2* in some of the samples. RI lies on chr19: 7,698,355-7,698,854; the upstream exon locates on chr19: 7,698,355-7,698,409 and the downstream exon locates on chr19: 7,698,740-7,698,854 [see Additional file 8: Table S6]. Compared to the mild MPP group, the severe MPP group had less RI spliced transcript events of *FCER2*, which could be an explanation for the up-regulation of the *FCER2* in severe MPP children (Fig. 4a).

*FCRLA* locates on chromosome 1q23.3 (chr 1:161,706,972-161,714,352). This gene encodes a protein similar to Fc receptor of gamma immunoglobulin (IgG), which is selectively expressed in B cells and may be involved in their development. Alternatively, spliced transcript variants of *FCRLA* that encode different protein isoforms have been found in the current study. Two exons of *FCLRA* were involved in mutual exclusive exon (MEX), the first exon starts at 161,710,759 and ends at 161,710,912 and the second exon starts at 161,711,207 and ends at 161,711,474 [see Additional file 9: Table S7]. The analysis of alternative splicing events indicated that the MEX frequency of the first exon in the severe MPP group was significantly higher than that in the mild MPP group, which may result in the down-regulated expression of *FCRLA* in severe MPP children (Fig. 4b).

## Discussion

Recent developments in RNA sequencing technology enabled elaborate analysis of gene expression in numerous human diseases. However, to our best knowledge, no report of RNA-sequencing study on human MPP has been published yet. The current study provides extensive information on gene expression and alternative splicing in the BALF of MPP children through transcriptome analysis, which is crucial for understanding the pathogenesis of severe MPP. The gene expression analysis revealed 14 up-regulated genes and 34 down-regulated genes in severe MPP children comparing to mild MPP children. The top 10 most up-regulated genes are *IGHV1-69*, *CH17-472G23.1*, *ATP1B2*, *FCER2*, *MUC21*, *IL13*, *FCRLB*, *CLEC5A*, *FAM124A*, and *INHBA* (Fig. 1a, Table 2). The top 10 most down-regulated genes are *OSTN-AS1*, *IL22RA2*, *COL3A1*, *C1orf141*, *IGKV2-29*, *RP11-731F5.2*, *IGHV4-4*, *KIRREL*, *DNASE1L3*, and *COL6A2* (Fig. 1a, Table 3).

Several key genes that are differentially expressed between severe MPP and mild MPP are associated with hyper-immune response and signaling. For example, IL13, a cytokine secreted by T helper type 2 (Th2) cells, can induce many features of allergic lung disease including airway hyper-responsiveness, goblet cell metaplasia, and mucus hyper-secretion, which all contribute to airway obstruction [25, 26]. IL13 is found to cause mucin overproduction through STAT6/EGFR-FOXA2 signaling and mucus plugging formation in MP infection, which results in pulmonary atelectasis or consolidation [27]. These results prove that IL13 play an important role in the airway obstruction of severe MPP. In addition, Wu Q et al. [26] have reported that IL13 can restrain MP clearance by the suppression of Toll-like receptor 2 in mice. Therefore, high levels of IL13 may make severe MPP children lose the ability to eradicate MP from the lung in primary infection, resulting in longer lasting MP infection and a hyper-immune response [7]. Another gene associated with hyper-immune response is *FCER2. FCER2* is a B cell specific antigen and a low-affinity receptor for IgE. It has essential roles in B cell growth, differentiation, and the regulation of IgE production. On the basis of study with animal models, *FCER2* has been implicated in IgE-mediated allergic diseases and bronchial hyper-reactivity [28]. It has been proved that *FCER2* is involved in the pharmacogenetic basis for severe exacerbations in children with asthma [29]. Up-regulated *FCER2* in severe MPP children has been discovered in the present study. Similarly, increased IgE levels in the serum of MPP patients have been reported [30]. Therefore, further study will be needed to prove that up-regulated *FCER2* causes the bronchial inflammation and hyper-immune reactivity. Among the up-regulated genes in severe MPP comparing to mild MPP, *FLT1* may also associate with hyper-immune response. *FLT1* (fms-like tyrosine kinase 1) encodes a member of the vascular endothelial growth factor receptor (VEGFR) family. Wu WK et al. demonstrate that Th2-related cytokines, such as IL4 and IL13, could drive the expression of *FLT1* [31]. Moreover, Th2-related cytokines can promote VEGF release in the airway, and VEGF has been proposed to be associated with severe MPP [32, 33]. Therefore, atopic children may be more prone to develop severe pneumonia [34]. We tentatively put forward the hypothesis that *IL13*, *FCER2*, and *FLT1* may be associated with each other and all of them are involved in the pathogenesis of severe MPP.

Based on our data [see Additional file 4: Table S3] and published results [35], more than half of the nucleated cells in BALF of MPP children without other pathogen infection are macrophages. But it is still unclear how macrophages are involved in the pathogenesis of severe MPP. Clustering analysis of the differentially expressed genes reveals that the expression patterns of *IL13*, *FCER2*, *FLT1*, and *CLEC5A* are similar in the severe MPP group (Fig. 2). One possible mechanism of severe MPP is the overactivation of macrophage in innate immune response [7]. *CLEC5A* (C-type lectin domain family 5, member A) is expressed on alveolar macrophages; it has been demonstrated to mediate macrophage response and play roles in pro-inflammatory cytokine expression and airspace enlargement in a mice model of chronic obstructive pulmonary disease (COPD) [36]. Muro S et al. suggested that MP infection could be an independent risk factor for COPD in the general population [37]. *CLEC5A* encodes a member of the CTL/CTLD (C-type lectin/C-type lectin-like domain) superfamily, which family members play roles in inflammation and immune responses. Teng O et al. have revealed that *CLEC5A*-mediated enhancement of the inflammatory response in myeloid cells contributes to influenza’s pathogenicity in vivo [38]. We found significant higher expression levels of *CLEC5A* in the BALF of severe MPP comparing to that of mild MPP. Therefore, our results support the hypothesis that *CLEC5A* is involved in the pathogenesis of severe MPP through the overactivation of macrophage. Protein functional classification of the differentially expressed genes indicates that signaling molecules including *IL13*, *CLEC5A*, and *INHBA* are obviously increased in severe MPP comparing to mild MPP [see Fig. 1b, Additional file 6: Table S4]. *INHBA* (Inhibin beta A) encodes a member of the transforming growth factor superfamily. The encoded preproprotein is proteolytically processed to generate a subunit of the dimeric activin and inhibin protein complexes. Rheumatoid arthritis synovium fluid (RA-SF) promotes *INHBA* production as a pro-inflammatory cytokine from macrophages in vitro [39]. Similarly, *INHBA* is reported to be up-regulated in endometritis by Hoelker M et al. [40]. It is interesting that the current study finds increased levels of *INHBA* in severe MPP. The causal relationship between the up-regulation of *INHBA* and severe MPP requires further investigation.

It is still unclear how B cells are involved in the pathogenesis of severe MPP. In the current study, KEGG pathway enrichment analyses have revealed changes related to primary immunodeficiency in severe MPP patients, which involves *CD79A*, *AICDA*, *CD19*, and *TNFRSF13C* genes (Table 4, Fig. 3) [22]. *CD79A*, *AICDA*, *CD19*, and *TNFRSF13C* are related to the B cell antigen signaling pathway; lower expression of these genes can lead to the deficiency of B cell functions. *CD79A* encodes the Igα protein of the B cell antigen component, which is necessary for expression and function of the B cell antigen receptor. Defected *CD79A* has been discovered in immunodeficiency-related diseases [41]. Similarly, *AICDA* (activation-induced cytidine deaminase) encodes a RNA-editing deaminase, which is expressed in a B cell differentiation stage-specific fashion. *AICDA* is involved in somatic hyper-mutation, gene conversion, and class-switch recombination of immunoglobulin genes [42]. Defects in *AICDA* can cause autosomal recessive hyper-IgM immunodeficiency syndrome type 2 (HIGM2) [43]. *CD19* is a B cell-specific molecule, which serves as a major costimulatory molecule for amplifying B cell receptor (BCR) responses. Morbach H et al. have revealed that *CD19* is required for TLR9-induced B cell activation and CD19/PI3K/AKT/BTK is an essential axis integrating BCRs and TLR9 signaling in human B cells [44]. In addition, biallelic *CD19* gene mutations cause common variable immunodeficiency in human. BCR-induced B cell responses are impaired in most patients with common variable immunodeficiency. *TNFRSF13C* (tumor necrosis factor receptor superfamily member 13C) encodes a receptor for BAFF (B cell activating factor), which enhances B cell survival in vitro and regulates the peripheral B cell population. *TNFRSF13C* is a principal receptor required for BAFF-mediated mature B cell survival and it has been reported to be associated with common variable immunodeficiency [45]. In our study, significantly decreased expression levels of *CD79A*, *AICDA*, *CD19*, and *TNFRSF13C* have been observed in the BALF of the severe MPP group comparing to the mild MPP group. Comparing to the local reduction of *CD79A*, *AICDA*, *CD19*, and *TNFRSF13C* genes found in BALF, these patient’s immunoglobulins and lymphocyte profiles in the peripheral blood are normal, which means they do not have any systemic immunodeficiency disease [see Additional file 2: Fig. S1, Additional file 3: Table S2 ]. We have found these local changes related to primary immunodeficiency in the bronchoalveolar of severe MPP. Local B cell-related immunodeficiency may be involved in the pathogenesis of severe MPP comparing with mild MPP. Figure 3 shows how these molecules are involved in the pathway of B cell differentiation and immunoglobulin gene class switch; down-regulation of these molecules may restrain antibody production and make the MPP children lose the ability to eradicate MP from the lung, which leads to happen of severe MPP [7].

Alternative splicing events of genes are involved in the diversity of proteome as well as genome evolution, control of developmental processes, and physiological regulation of various biological systems [46]. It could be deduced that dysregulation of alternative splicing event is often linked to various human diseases [47]. However, alternative splicing events in the context of MPP have rarely been investigated. The current study discovered significant differential alternative splicing events in *FCER2* and *FCRLA* which could be an explanation for the differential expression of these two genes between severe MPP and mild MPP. The alternative splicing type of *FCER2* is retained intron (RI), which can change the function and expression levels of a gene [48]. Therefore, lower rate of RI in the severe MPP group comparing to the mild MPP group may partly explain the significantly increased expression levels of *FCER2* in the severe MPP group. *FCRLA* is a soluble resident endoplasmic reticulum protein. It is capable of associating with multiple Ig isotypes including IgM, IgG, and IgA, which makes it unique among the large family of Fc receptors. The expression of *FCRLA* is restricted to B lineage and is most abundant in germinal center B lymphocytes [49]. In the present study, the alternative splicing type of *FCRLA* is MEX, which is significantly increased in the severe MPP group comparing to the mild MPP group. According to the literatures [50, 51], alternative splicing may be a causal explanation for the down-regulation of *FCRLA* in the severe MPP group.

Previous studies have found that cell-mediated immune response, specifically Th1-type cytokines such as IL8, IL18, and IFNγ, plays important roles in the mechanism of MPP [9–11, 52]. Most of these studies observed serum levels of Th1 cytokines, but we have chosen BALF to study the local differentially expressed genes. Cytokines may be expressed differently in BALF comparing to that in the peripheral blood; this could partly explain why significant difference of IL8, IL18, and IFNγ was not found in the current study. Additionally, our transcriptome analysis focused on comparing severe MPP with mild MPP in order to study the specific pathogenesis of severe MPP; similar results have been reported by Kang YM et al. group [53]. In their study, higher levels of IFNγ in BALF of MPP patients comparing with that of control children were discovered, but no significant difference of IFNγ was found between the severe MPP group and mild MPP group.

Some limitations of the current study should be discussed. First, this is a small size transcriptome analysis between mild MPP children and severe MPP children; bigger sized analysis would be preferred in future study to support solid conclusions. However, three samples in each group have been chosen carefully to receive next-generation sequencing separately; quality control analysis of the whole process has been greatly satisfied. Correlation matrix shows a high consistency of measurements within each group. Principal component analysis (PCA) shows good repeatability and correlation of these samples. Second, this is a preliminary study on the pathogenesis of severe MPP comparing to mild MPP. Further study will be needed to further prove the hypothesis that gene reduction related to primary immunodeficiency in bronchoalveolar is involved in the pathogenesis of severe MPP. Patient’s primary cell culture and in vivo experiments using animal model may help to clarify how these genes and pathways are involved in the pathogenesis of severe MPP in the future.

## Conclusions

The current study presented gene expression profiles as well as alternative splicing in BALF samples from MPP patients by next-generation RNA sequencing. This study clearly indicates that the up-regulation of *IL13*, *FCER2*, *FLT1*, and *CLEC5A* and the down-regulation of *CD79A*, *AICDA*, *CD19*, and *TNFRSF13C* may contribute to the pathogenesis of severe MPP or the progression from mild MPP to severe MPP. Furthermore, the differentially expression of *FCER2* and *FCRLA* may be due to the alternative splicing; further studies will be required to confirm this hypothesis.

## Additional files

Additional file 1: Table S1. Summary of patients’ information. (XLS 30 kb)
Additional file 2: Figure S1. The levels of immunoglobulins in the peripheral blood of MPP patients. A. The levels of IgG in each patients. Normal range of 5.09–14.17 g/L is shown as dotted lines. B. The levels of IgA in each patients. Normal range of 0.31–1.92 g/L is shown as dotted lines. C. The levels of IgM in each patient. Normal range of 0.98–2.56 g/L is shown as dotted lines. (TIF 611 kb)
Additional file 3: Table S2. Lymphocyte profiles in the peripheral blood of the MPP patients. (XLS 29 kb)
Additional file 4: Table S3. Nucleated cell count in the BALF of the MPP patients. (XLS 30 kb)
Additional file 5: Figure S2. Evaluation of each sample included in the current study. A. The correlation coefficient heat map. Correlation matrix shows a high consistency of measurements within each group. *R* ^2^ ≥ 0.8 is needed for the up-coming analyzing. B. Principal component analysis (PCA) plot. PCA is conducted to evaluate the clustering nature of the samples. Each point represents one sample. The repeatability of the samples has been shown. (TIF 5292 kb)
Additional file 6: Table S4. Protein functional classification in differentially expressed genes between severe MPP and mild MPP. (XLS 32 kb)
Additional file 7: Table S5. Summary of the differential expressed genes with skipped exon. (XLS 37 kb)
Additional file 8: Table S6. Summary of the differential expressed genes with retained intron. (XLS 28 kb)
Additional file 9: Table S7. Summary of the differential expressed genes with mutually exclusive exon. (XLS 30 kb)
Additional file 10: Table S8. Summary of the differential expressed genes with alternative 5' splice site. (XLS 28 kb)
Additional file 11: Table S9. Summary of the differential expressed genes with alternative 3' splice site. (XLS 29 kb)

## Abbreviations

AICDA: Activation-induced cytidine deaminase
BAFF: B cell activating factor
BAL: Bronchoalveolar lavage
BALF: Bronchoalveolar lavage fluid
BCR: B cell receptor
CAP: Community acquired pneumonia
CLEC5A: C-type lectin domain family 5, member A
COPD: Chronic obstructive pulmonary disease
CTL: C-type lectin
CTLD: C-type lectin-like domain
DNA: Deoxyribonucleic acid
ELISA: Enzyme-linked immunosorbent assay
FDR: False discovery rate
IgA: Immunoglobin A
IgG: Immunoglobin G
IgM: Immunoglobin M
IGV: Integrative Genomics Viewer
Igα: Immunoglobin α
KEGG: Kyoto Encyclopedia of Genes and Genomes
MEX: Mutual exclusive exon
MP: *Mycoplasma pneumoniae*
MPP: *Mycoplasma pneumoniae* pneumonia
PANTHER: Protein analysis through evolutionary relationships
PCR: Polymerase chain reaction
RA-SF: Rheumatoid arthritis synovium fluid
RI: Retained intron
RNA: Ribonucleic acid
Th2: T helper type 2
TNFRSF13C: Tumor necrosis factor receptor superfamily member 13C
