# Supplementary figures and images for: Transcriptome analysis of bronchoalveolar lavage fluid from children with severe Mycoplasma pneumoniae pneumonia reveals novel gene expression and immunodeficiency
Source: Hum Genomics. 2017 Mar 16;11:4. doi: 10.1186/s40246-017-0101-y (PMC5356355; doi:10.1186/s40246-017-0101-y)

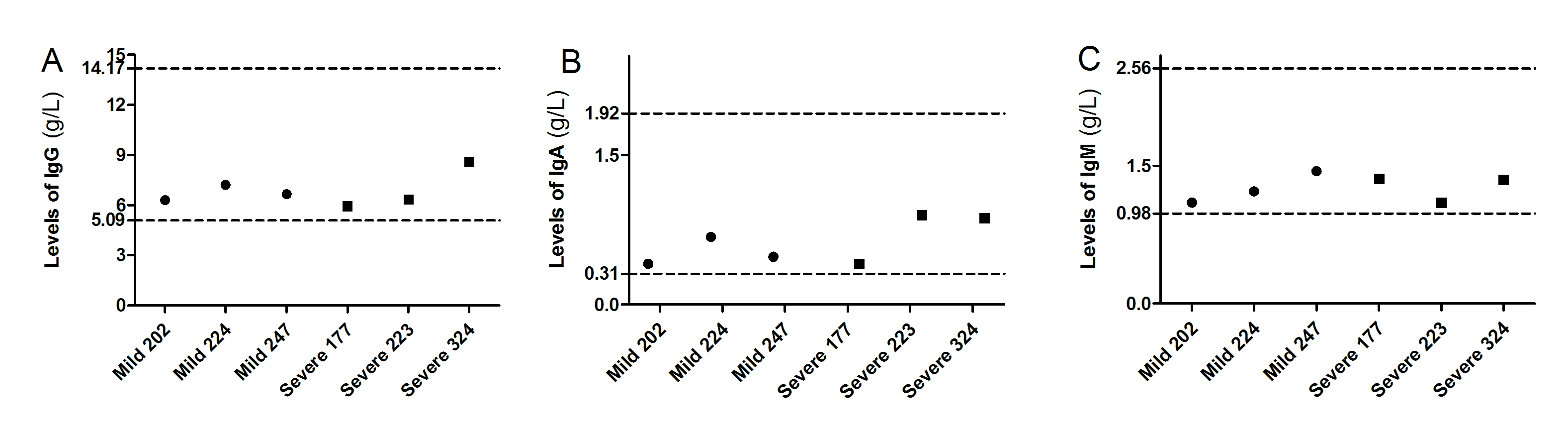

Supplement: Additional file 2: Figure S1. — The levels of immunoglobulins in the peripheral blood of MPP patients. A. The levels of IgG in each patients. Normal range of 5.09–14.17 g/L is shown as dotted lines. B. The levels of IgA in each patients. Normal range of 0.31–1.92 g/L is shown as dotted lines. C. The levels of IgM in each patient. Normal range of 0.98–2.56 g/L is shown as dotted lines. (TIF 611 kb) [file 40246_2017_101_MOESM2_ESM.tif]

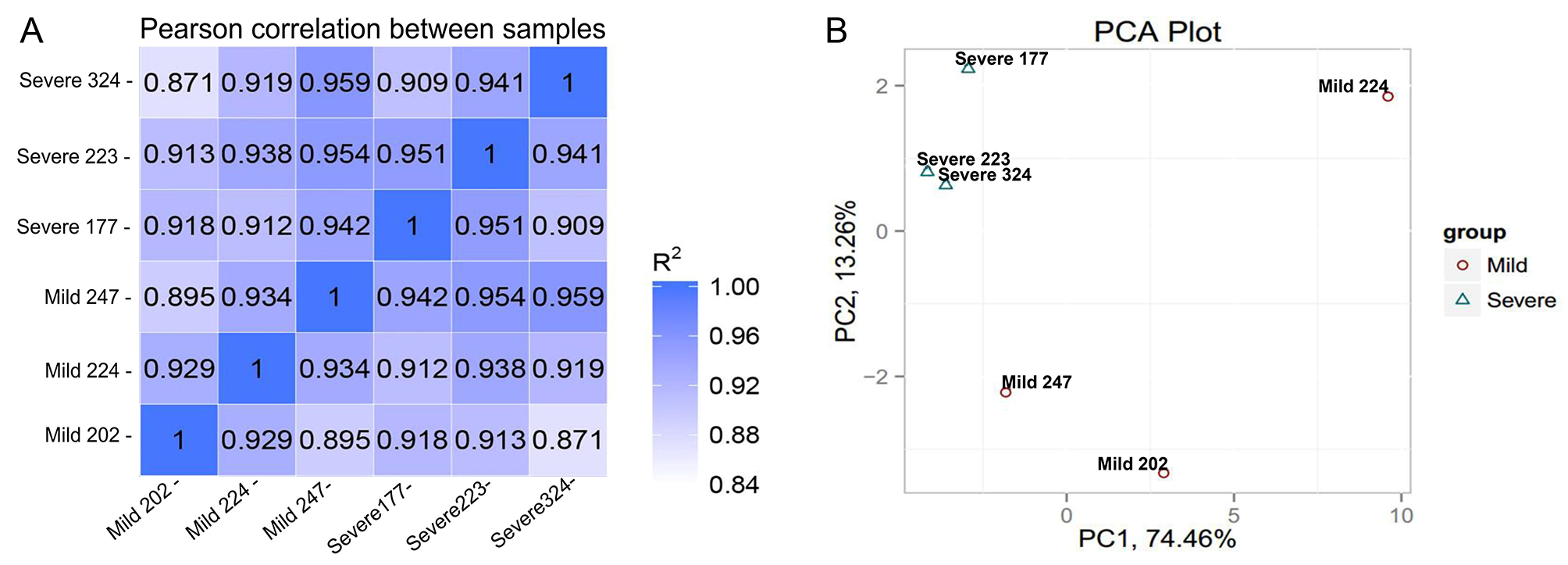

Supplement: Additional file 5: Figure S2. — Evaluation of each sample included in the current study. A. The correlation coefficient heat map. Correlation matrix shows a high consistency of measurements within each group. R 2 ≥ 0.8 is needed for the up-coming analyzing. B. Principal component analysis (PCA) plot. PCA is conducted to evaluate the clustering nature of the samples. Each point represents one sample. The repeatability of the samples has been shown. (TIF 5292 kb) [file 40246_2017_101_MOESM5_ESM.tif]
